# Strategies for reducing police arrest in the context of an HIV prevention programme for female sex workers: evidence from structural interventions in Karnataka, South India

**DOI:** 10.7448/IAS.19.4.20856

**Published:** 2016-07-18

**Authors:** Parinita Bhattacharjee, Shajy Isac, Leigh M McClarty, Haranahalli L Mohan, Srinath Maddur, Sunitha B Jagannath, Balasubramanya K Venkataramaiah, Stephen Moses, James F Blanchard, Vandana Gurnani

**Affiliations:** 1 Karnataka Health Promotion Trust, Bangalore, India; 2 Department of Community Health Sciences, University of Manitoba, Winnipeg, MB, Canada

**Keywords:** female sex worker, FSW, police, arrest, HIV prevention, India, HIV/AIDS, violence, structural intervention

## Abstract

**Introduction:**

Female sex workers (FSWs) frequently experience violence in their work environments, violating their basic rights and increasing their vulnerability to HIV infection. Structural interventions addressing such violence are critical components of comprehensive HIV prevention programmes. We describe structural interventions developed to address violence against FSWs in the form of police arrest, in the context of the Bill and Melinda Gates Foundation's India AIDS Initiative (*Avahan*) in Karnataka, South India. We examine changes in FSW arrest between two consecutive time points during the intervention and identify characteristics that may increase FSW vulnerability to arrest in Karnataka.

**Methods:**

Structural interventions with police involved advocacy work with senior police officials, sensitization workshops, and integration of HIV and human rights topics in pre-service curricula. Programmes for FSWs aimed to enhance collectivization, empowerment and awareness about human rights and to introduce crisis response mechanisms. Three rounds of integrated behavioural and biological assessment surveys were conducted among FSWs from 2004 to 2011. We conducted bivariate and multivariate analyses using data from the second (R2) and third (R3) survey rounds to examine changes in arrests among FSWs over time and to assess associations between police arrest, and the sociodemographic and sex work-related characteristics of FSWs.

**Results:**

Among 4110 FSWs surveyed, rates of ever being arrested by the police significantly decreased over time, from 9.9% in R2 to 6.1% in R3 (adjusted odds ratio (AOR) [95% CI]=0.63 [0.48 to 0.83]). Arrests in the preceding year significantly decreased, from 5.5% in R2 to 2.8% in R3 (AOR [95% CI]=0.59 [0.41 to 0.86]). FSWs arrested as part of arbitrary police raids also decreased from 49.6 to 19.5% (AOR [95% CI]=0.21 [0.11 to 0.42]). Certain characteristics, including financial dependency on sex work, street- or brothel-based solicitation and high client volumes, were found to significantly increase the odds of arrest for participants.

**Conclusion:**

Structural interventions addressing police arrest of FSWs are feasible to implement. Based on our findings, the design of violence prevention and response interventions in Karnataka can be tailored to focus on FSWs, who are disproportionately vulnerable to arrest by police. Context-specific structural interventions can reduce police arrests, create a safer work environment for FSWs and protect fundamental human rights.

## Introduction

Effective HIV prevention programmes for female sex workers (FSWs) must address violence that is commonly experienced in their work environments [1]. Modelling exercises recently conducted in India, Canada and Kenya – countries in which relatively large proportions of FSWs report experiences of sexual violence – show that a reduction in or elimination of sexual and physical violence and its consequences would substantially avert HIV infection among FSWs and their clients over the next 10 years [2]. Globally, the rights of FSWs are violated due to experiences of violence and harassment by police and clients [3–10], stigma and discrimination when seeking health services [9–11] and other forms of structural violence [12,13] that threaten their well-being and livelihood. These violations directly and indirectly increase vulnerability to HIV and undermine HIV prevention efforts [14,15].

Although violence against FSWs at the hands of clients, partners, pimps and madams is widely cited in literature from India [3,6,7,12,16–20], state actors (e.g., police or other law enforcement officers) are also common perpetrators of violence and harassment [1,10,17,21,22]. Police can exert tremendous power over FSWs through the use of physical violence, harassment, unlawful arrests, forced detainment and forced or free sex, particularly in countries where sex work is criminalized [1,7,9,23]. Notably, Beattie *et al*. [17] recently reported a significant association between police arrest and increased HIV prevalence among FSWs in Karnataka. For some FSWs, debts that accumulate from bail payments, court fees and legal costs following arrest tend to result in heightened financial insecurity and hardship [18]. In Karnataka, these debts have been shown to increase HIV vulnerability, as FSWs will often settle for “riskier” agreements with clients that yield higher per-act earnings [10]. Police seizure of condoms as evidence of sex work has also been shown to increase HIV risk among FSWs through higher rates of unprotected sex [8,23–25]. These seizures interfere with the operation of peer-led HIV prevention programmes, as peer educators and sex work venue (e.g., bar, lodge) managers fear carrying or stocking condoms for distribution [26,27]. Furthermore, fear of arrest and extortion by police can force FSWs to move to different geographic locations [1], thus disrupting important social support networks, creating barriers for programme outreach and limiting access to HIV prevention tools and services [1,27,28]. Geographic displacement may also compel FSWs to work in unsafe environments, forego condom negotiation and engage in unprotected sex [29,30]. These negative experiences ultimately undermine FSWs’ willingness and ability to seek out and obtain protection from police, while creating environments of impunity for violent clients, offending police officers and other perpetrators [23,31]. Importantly, the environment of fear and anxiety created by these policing practices has also been associated with increased vulnerability to HIV infection [5].

### Context

The southern state of Karnataka, India, has a population of approximately 60 million, and findings from epidemiological mapping estimate that approximately 130,000 FSWs live and work in the state [32]. Although HIV prevalence among Karnataka's general population is low, at 0.5%, FSWs are disproportionately burdened by the HIV epidemic, with an estimated overall prevalence of 5.1% in 2014 [33]. Furthermore, substantial regional heterogeneity exists across the state [34], with an estimated HIV prevalence among FSWs in some districts greater than 25% [35]. Although the buying and selling of sex is not illegal in India *per se*, the Immoral Traffic (Prevention) Act of 1956 (ITPA) [36] and its 2006 amendment [37] criminalize most aspects of sex work [38,39]. The primary goal of the ITPA in its current form is to address issues related to trafficking; the law prohibits any person from directly or indirectly benefitting from prostitution (Sections 3 and 4), buying sex (Sections 5 and 6) or selling sex in the vicinity of a public place (Section 7) [36,38]. The ITPA also stipulates that any space in close proximity to any public place that is being used for the buying or selling of sex shall be closed and all occupants evicted (Section 18) [36]. The majority of FSWs in Karnataka are street- and home-based, with the former largely concentrated in the southern regions of the state and the latter more common in northern districts [40]. As such, police in Karnataka most often arrest FSWs under Sections 7 and 18 of the ITPA.

The University of Manitoba and the Karnataka Health Promotion Trust (KHPT) were lead implementing partners in Karnataka for *Avahan –* the India AIDS Initiative of the Bill and Melinda Gates Foundation – from 2003 to 2013 [17,41,42]. HIV prevention programmes were initiated in 18 of Karnataka's 30 districts, reaching approximately 60,000 FSWs, and by 2013 the interventions were successfully handed over to the government of Karnataka and local FSW community-based organizations (CBOs) [4]. The initial design of the HIV prevention programmes mainly focused on reaching FSWs in Karnataka with HIV prevention information through a peer educator scheme; increasing condom distribution; improving accessibility of HIV testing, STI testing and STI treatment; and facilitating linkage to HIV care for anyone testing positive. However, consultations with FSWs at the beginning of programme implementation highlighted violence as a common and significant challenge in the daily lives of FSWs in Karnataka [3]. In particular, FSWs in Karnataka identified intimate partners, clients, madams/pimps, rowdies and police as the primary perpetrators of violence [17]. Importantly, previous work indicates that FSWs who report experiencing violence within the past year are significantly less likely to access HIV prevention services and report condom use with clients, in part due to the negative impact violence has on FSWs’ mental health [3]. In response, KHPT, alongside partnering organizations, including sex worker collectives, developed two structural interventions within the larger HIV prevention programme. The interventions were designed to create an environment in which FSWs would be able to more readily address violence experienced at the hands of various perpetrators, including police.

In this paper, we describe, for the first time, the structural interventions that were developed with FSW communities to reduce rates of police arrest among FSWs in Karnataka. We use data from integrated behavioural and biological assessment (IBBA) surveys conducted at programme sites to examine trends in arrests over time, as well as associations between FSW characteristics and arrest, highlighting the need to focus on FSWs who are disproportionately vulnerable to police arrest.

## Methodology

### Survey design and sampling

A series of district-level, cross-sectional IBBA surveys were conducted among random samples of FSWs in four districts of Karnataka (Belgaum, Bellary, Shimoga and Bangalore). Districts were chosen purposively based on geographic heterogeneity, variation in regional HIV prevalence [34] and predetermined estimates of the FSW population in each district. The sample size for each IBBA survey comprised approximately 60% of the total estimated FSW population in each district. Initial estimates of FSW population size per district were obtained through epidemiological mapping in 2004 [43] and were subsequently validated through annual monitoring and evaluation exercises for *Avahan*. Programmes were initiated in each district between April 2004 and October 2005, with Round 1 (R1) surveys conducted from August 2005 through July 2006, Round 2 (R2) from July 2008 through January 2009 and Round 3 (R3) from September 2010 through August 2011.

Sample size calculations and sampling methodology have been previously described in detail [44]. The target sample size per district was fixed at 400 completed surveys, except for Bangalore, where the sample size was increased to 800 to better represent the diversity of FSW typologies and the large FSW population in this urban metropolis. Two different probability-based sampling approaches were adopted. Conventional cluster sampling [45] was used to sample FSWs practising out of homes or brothels, where the population sizes were relatively stable. Sampling street-based FSWs involved time-location cluster (TLC) sampling methods [45], in which intervention districts were divided into several TLCs and then a predetermined number of clusters were randomly selected for inclusion in the study sample. FSWs who were sampled from their home, the street or another public place, or a brothel were asked where they “most often” picked up clients. Participants indicating that they most often solicited via phone were classified as phone-based sex workers in these analyses. However, FSWs who exclusively used mobile phones to pick up clients were excluded from our sample, as recruitment was not done over the phone, and there are currently no epidemiological data available from Karnataka to estimate the size of the phone-based FSW population in study districts.

The IBBAs were designed to be culturally sensitive and context-specific, as previously described [35,46]. Only the tools used in R2 and R3 contained questions on police arrest experienced by FSWs, yielding the data presented in this paper. All surveys were administered in person by trained interviewers in the local language, Kannada. To ensure confidentiality, no identifying information was collected, which precluded data linkage between the R2 and R3 surveys.

### Statistical analyses

Statistical analyses were performed with SPSS v22.0. Appropriate weights were used to account for the differential recruitment of FSWs by typology within districts, non-response rates, and probabilities of selection across districts. The general weighting procedure for IBBA required two steps. First, a cluster weight was calculated and a cluster-level non-response adjustment was applied to the cluster weight; it was calculated independently for each design domain (type of sex work site). Second, an FSW weight was calculated and an FSW-level non-response adjustment was applied to the FSW weight. The FSW weight was calculated independently for each cluster. The overall sampling weight attached to each FSW record is the product of the cluster weight and the FSW weight. These final weights were normalized so that the total number of weighted cases equalled the total number of unweighted cases.

Both bivariate and multivariate analyses were performed to assess the association between police arrest and participant characteristics (sociodemographic and sex work-specific). Multivariate analyses were used to adjust for potential confounding factors by district, survey round, and sociodemographic and sex work characteristics. The dichotomous outcome variables of interest, “ever been arrested” and “arrested in the past year,” were analyzed using logistic regression models, adjusted for participants’ districts of interview and origin, age, self-reported literacy, involvement in work besides sex work, sex work typology, sex work duration, average number of clients per week, marital status, age at first sex, age at start of sex work, duration of relationship with main partner and years since first contact with an HIV prevention programme.

### Ethical considerations

The Institutional Ethical Review Board of St. John's National Academy of Health Sciences, Bangalore, India, and the Health Research Ethics Board at the University of Manitoba, Winnipeg, Canada, approved the study. Verbal informed consent was obtained, in the presence of a witness, from all survey participants. Statutory approval for the conduct of the IBBA surveys and their protocols was obtained from the government of India's Health Ministry Screening Committee.

### Description of the intervention programmes

**Figure 1:**
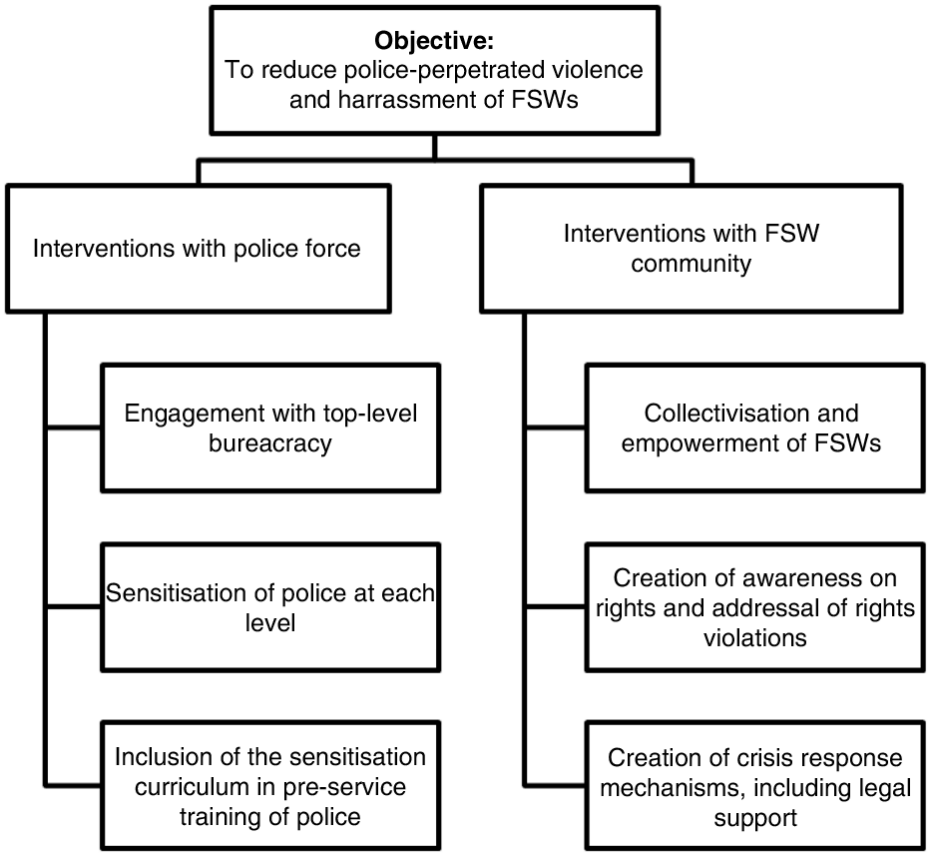
Two-pronged strategy to address police violence against female sex workers in Karnataka, South India.

One key objective of the structural interventions designed and implemented for the programme was to reduce police violence and harassment experienced by FSWs. A two-pronged strategy was employed (Figure 1). The first prong involved advocacy and sensitization work with police, on behalf of FSWs, starting from the highest level of bureaucracy within the Karnataka State Police. The second prong focused on capacity building and collectivization strategies among FSWs, which aimed to better equip communities to challenge police violence through collective empowerment [42].

### Interventions with police

In 2004, the programme initiated its advocacy work with police by engaging with top-level civil servants in Karnataka's Ministry of Home Affairs [47]. This resulted in the Director-General of Police issuing instructions to all police personnel to interpret the ITPA in a way that reduces harm towards FSWs and penalizes traffickers and abusive pimps/madams rather than FSWs [48]. The Director-General also made a commitment that any allegation of police harassment or connivance would undergo prompt enquiry and strict action [48].

Next, one-day sensitization workshops [49] were held with all cadres of police personnel at all police stations within the programme districts. These workshops were conducted by local implementing agencies, government officials in charge of managing HIV-related health programmes in the districts, senior police officials, human rights experts and members of local FSW communities [48]. Multiple sensitization workshops were held to build rapport with the local police and accommodate frequent turnover in police personnel. During the workshops, officers were guided through the interpretation of existing laws that were being used to persecute FSWs; educated about fundamental human rights and consequences of violating those rights, with specific reference to local sex work communities; and informed about the lives of FSWs and their daily challenges. Police officers were also provided with evidence that explicitly links violence and harassment directed towards FSWs with poor health outcomes. Between 2005 and 2011, 85 senior police officials and 13,594 police officers were trained as facilitators, covering 60% of all members of the state police force.

Post-sensitization, the implementing partners regularly followed up with the police stations to maintain rapport with the officers and invited police officials to attend a variety of programme activities as special guests, including inaugural events for programme drop-in centres and clinics. This helped foster mutually beneficial working relationships between the police, implementing agencies and the FSWs themselves. In addition, facilitators worked with police training academies to incorporate sessions on sex work and the ITPA into pre-service and in-service curricula, to sustain programme efforts.

### Interventions with FSWs

In 2006, the violence reduction programme expanded to include interventions with FSWs. A central component of the interventions with FSWs was community collectivization [50]. This step led to the establishment of support groups within each programme site and collectives at the district and sub-district levels, while fostering solidarity for collective action. This resultant “collective empowerment” [42] leads to greater autonomy and reduces experiences of violence and coercion among FSWs. Previous findings from *Avahan* programmes have shown that FSWs who are members of any peer group experience significantly less violence than non-peer group members [51].

As such, a series of capacity-building sessions [49] were organized with FSWs to clarify their rights, as protected by the Indian constitution and national laws, to familiarize FSWs with existing laws under which they can be arrested and to facilitate their interpretation. As part of these capacity-building exercises, FSWs were taken to police stations to review the procedures for registering complaints against perpetrators of violence, and face-to-face interactions between FSWs and officers were organized to initiate an open dialogue.

Finally, crisis response and management systems were set up in all intervention districts to respond to any violence reported by FSWs. Dedicated 24-hour phone lines were established, with telephone numbers distributed widely within the community to encourage FSWs to call and seek support in case of crisis or violence. Each crisis management system also included a 24-hour crisis management team [3], including a human rights lawyer, who provided legal counsel when the crisis management team dealt with issues such as providing bail to fellow FSWs and participating in court cases involving community members.

## Results

In total, 4110 FSWs were interviewed over two rounds of IBBA surveys, which were conducted 20 months apart – 48% in R2 and 52% in R3 (Table 1). Sixteen percent of survey participants were young (<25 years), about two-thirds reported illiteracy, and another two-thirds reported working in jobs besides sex work (e.g., domestic labour, hawking, petty shopkeeping and fruit/vegetable vending). All participants were citizens of India and identified as ethnically Indian (data not shown). Twenty-one percent of participants reported practicing sex work somewhere besides their home district, of whom only 6% were not natives of Karnataka (data not shown). FSWs most commonly (44%) solicited clients in the street or other public places. Seventeen percent of participants were relatively new to sex work (practising for <2 years), whereas 51% reported involvement in sex work for more than five years. One-quarter of participants entered into sex work between the ages of 20 and 24 years, while 21% started sex work before they were 20 years old. Nearly one-half of FSWs reported at least 10 clients in an average week. In R2 and R3, most participants reported at least one year of exposure to HIV prevention services through a CBO or non-governmental organization.

**Table 1:** Sociodemographic and sex work-related characteristics of FSW participants in Karnataka, by survey round

		IBBA survey round		
			
	Total	R2	R3	
				
	*(N=*4110)	(*n=*1953)	(*n=*2157)	
	%	%	%	*p*
District				
Belgaum	28.0	25.6	30.2	
Bellary	23.1	24.2	22.1	
Shimoga	23.5	23.6	23.5	
Bangalore Urban	25.4	26.7	24.3	0.002
Age (years)				
< 25	15.7	17.8	13.8	
≥ 25	84.3	82.2	86.2	<0.001
Literacy				
Literate	35.3	34.8	35.8	
Illiterate	64.7	65.2	64.2	0.670
Work other than sex work				
Yes	64.7	62.4	66.7	
No	34.8	36.8	33.0	<0.001
Location of sex work practice				
Practised sex work within home district	79.5	78.4	80.6	
Practised sex work outside of home district	20.5	21.6	19.4	0.002
Usual place of solicitation				
Home	27.3	37.3	18.5	
Street/public place	44.5	44.1	44.9	
Brothel	7.0	11.0	3.5	
Phone	21.1	7.6	33.2	<0.001
Years practising sex work				
≤ 2	16.9	19.6	14.4	
3 to 4	32.1	30.3	33.7	
≥ 5	51.0	50.1	51.9	<0.001
Number of clients in an average week				
< 10	52.2	42.4	60.9	
≥ 10	47.8	57.6	39.1	<0.001
Age at start of sex work (years)				
≤ 19	21.5	23.8	19.5	
20 to 24	24.9	25.0	24.8	
25 to 29	24.2	23.7	24.6	
30 to 34	17.4	16.9	17.7	
≥ 35	12.1	10.6	13.3	<0.001
First contact with/by CBO or NGO				
Not aware of CBO/NGO	3.4	4.5	2.5	
< 1 year ago	12.5	15.5	9.9	
1 to 2 years ago	34.3	37.7	31.3	
3 to 4 years ago	31.7	33.5	30.1	
≥ 5 years ago	18.0	8.8	26.2	<0.001

CBO, community-based organization; FSW, female sex worker; IBBA, integrated behavioural and biological assessment; NGO, non-governmental organization

### Police arrest of FSWs in intervention districts

Table 2 presents the changes in FSW arrest over time. Overall, 9.9% of FSWs reported ever being arrested in R2, and this proportion significantly decreased to 6.1% in R3. Although 5.5% percent of R2 participants reported experiencing arrest in the preceding year, this number decreased significantly to 2.8% in R3. Among the FSWs surveyed in R2, nearly half reported that they had been arrested as part of an arbitrary, routine police raid and this proportion significantly decreased to 19.5% in the subsequent survey.

**Table 2:** Changes in police arrest among FSWs in intervention districts over time^a^

	Ever arrested			Arrested in the last year			Arrested during a routine raid		
			
IBBA survey round	%	AOR (95% CI)	*p*	%	AOR (95% CI)	*p*	%	AOR (95% CI)	*p*
R2 (*n=*1953)	9.9	Ref.	–	5.5	Ref.	–	49.6	Ref.	–
R3 (*n=*2157)	6.1	0.63 (0.48 to 0.83)	<0.001	2.8	0.59 (0.41 to 0.86)	0.006	19.5	0.21 (0.11 to 0.42)	<0.001

a Models adjusted for participant characteristics, as previously described; AOR, adjusted odds ratio; FSW, female sex worker; IBBA, integrated behavioural and biological assessment

### Peer support received by FSWs following arrest

Exposure to the structural interventions over time is shown in Table 3. A significantly higher proportion of women received support from fellow FSWs following arrest at R3 (69.6%), when compared to R2 (40.7%).

**Table 3:** Changes in post-arrest peer support received by FSWs in intervention districts over time^a^

	Received peer support following arrest		
	
IBBA survey round	%	AOR (95% CI)	*p*
R2 (*n*=1953)	40.7	Ref.	–
R3 (*n*=2157)	69.6	5.21 (2.83 to 9.60)	<0.001

a Models adjusted for participant characteristics, as previously described; AOR, adjusted odds ratio; FSW, female sex worker; IBBA, integrated behavioural and biological assessment

### Sociodemographic and sex work-related characteristics associated with police arrest

To better understand whether certain FSWs are disproportionately vulnerable to police arrest and harassment, we examined the relationships between history of arrest and sociodemographic and sex work-related characteristics (Table 4). FSWs who were illiterate or practising sex work outside of their home district had significantly greater odds of being arrested than literate or local FSWs, ever and in the last year (*p<*0.05). The odds of ever being arrested and of being arrested in the last year were significantly higher for participants who relied solely on sex work for income. When compared to home-based FSWs, women who solicited clients in brothels or the street were significantly more likely to have ever been arrested, but only street-based FSWs were more likely to have been arrested in the past year. Additionally, women entertaining ≥10 clients/week had over two times the odds of being arrested, ever and in the last year, when compared to participants reporting <10 clients/week. Women who started sex work when they were ≥25 years old had lower odds of ever being arrested, when compared to women who entered sex work at ≤19 years old, but age at entry was not a significant predictor of arrest in the last year.

**Table 4:** Influence of select sociodemographic and sex work-related characteristics on FSWs’ histories of arrest^a^

		Ever arrested			Arrested in the last year		
			
	*n*	%	AOR (95% CI)	*p*	%	AOR (95% CI)	*p*
Age (years)							
< 25	1045	11.8	Ref.	–	5.1	Ref.	–
≥ 25	809	7.0	1.47 (0.95 to 2.27)	0.081	4.4	0.79 (0.46 to 1.36)	0.403
Literacy							
Literate	1546	6.0	Ref.	–	3.3	Ref.	–
Illiterate	2564	9.0	1.44 (1.09 to 1.90)	0.011	4.5	1.49 (1.03 to 2.16)	0.034
Work other than sex work							
Yes	2607	5.3	Ref.	–	2.7	Ref.	–
No	1488	12.7	2.00 (1.56 to 2.56)	<0.001	6.6	1.74 (1.24 to 2.44)	<0.001
Location of sex work practice							
Practised sex work within home district	3157	7.3	Ref.	–	3.4	Ref.	–
Practised sex work outside of home district	953	10.4	1.61 (1.19 to 2.19)	0.002	6.7	1.78 (1.20 to 2.62)	0.004
Usual place of solicitation							
Home	1702	9.8	Ref.	–	5.3	Ref.	–
Street/public place	1150	4.4	2.27 (1.61 to 3.20)	<0.001	2.3	2.22 (1.40 to 3.53)	<0.001
Brothel	316	18.6	3.01 (1.90 to 4.77)	<0.001	8.8	1.759 (0.94 to 3.30)	0.079
Phone	941	4.8	1.56 (0.97 to 2.50)	0.064	2.3	1.41 (0.73 to 2.70)	0.306
Years practising sex work							
≤ 2	755	6.0	Ref.	–	5.3	Ref.	–
3 to 4	1391	6.0	1.15 (0.73 to 1.80)	0.561	3.8	0.931 (0.56 to 1.56)	0.787
≥ 5	1964	9.7	1.16 (0.70 to 1.92)	0.563	3.9	0.78 (0.43 to 1.43)	0.425
Number of clients in an average week							
< 10	2189	4.1	Ref.	–	2.0	Ref.	–
≥ 10	1921	12.1	2.27 (1.72 to 3.00)	<0.001	6.3	2.27 (1.55 to 3.32)	<0.001
Age at start of sex work (years)							
≤ 19	833	12.1	Ref.	–	6.0	Ref.	–
20 to 24	1031	8.7	0.78 (0.54 to 1.13)	0.192	4.4	0.80 (0.49 to 1.31)	0.375
25 to 29	1024	6.7	0.59 (0.38 to 0.91)	0.018	3.5	0.77 (0.42 to 1.41)	0.392
30 to 34	727	5.8	0.58 (0.36 to 0.91)	0.027	3.7	0.95 (0.50 to 1.79)	0.870
≥ 35	495	4.2	0.43 (0.24 to 0.77)	0.005	1.8	0.46 (0.20 to 1.08)	0.076

a Models adjusted for participant characteristics, as previously described; AOR, adjusted odds ratio; FSW, female sex worker; IBBA, integrated behavioural and biological assessment

## Discussion

This paper is the first to focus specifically on the factors associated with police arrest among FSWs in Karnataka. We demonstrate that the implementation of structural interventions involving police may contribute to reducing rates of arrest over time and highlight the disproportionate vulnerability of specific FSWs in Karnataka to police violence and arrest.

Importantly, we provide an in-depth description of the design and implementation of structural interventions that may play a role in reducing police arrest among FSWs in Karnataka. The primary intent of this paper is to provide guidance and direction to others wishing to develop similar programmes aimed at reducing police violence and arrest targeted at FSWs in other geographic and epidemiological contexts. There are many emerging examples of structural interventions directed at police in the context of HIV prevention programmes for FSWs, even in countries where sex work is criminalized [20]. Such programmes create a better understanding of the sex trade among police officers, improve access to health and social services for FSWs and have shown a clear reduction in police violence toward FSWs [20]. Our findings bolster growing evidence that even in an environment such as in India, where sex work is criminalized, structural approaches to address violence can be effectively delivered to scale, to reduce arrest of and violence against FSWs [3,6,10,17].

Police reform is a key element for enhancing partnerships between police and communities of FSWs, especially in an environment that supports violence, arbitrary arrest and harassment of FSWs [20]. Various police-focused strategies have been employed by programmes in an attempt to enhance the enabling environment for FSWs. Police training and education is one of the most common approaches, as exemplified by Save the Children's *Poro Sapot* programme in Papua New Guinea, which focuses on creating safe environments within which commercial interactions can take place [52]. Another strategy employed by Keeping Alive Societies Hope (KASH) in Kenya since 2007 is based upon conducting joint workshops with police officers and FSWs. Due to its success, KASH is now being supported by police training centres across Nyanza Province [53]. As described, our strategy was to attempt to reduce police violence through sensitization workshops and programmes that built capacity and collective empowerment [42] among FSW communities. Based on our findings and previous work [22], there is now convincing evidence that this kind of structural intervention can lead to significant reductions in police harassment and violence and better equip FSWs to address such violence.

This study also highlights the variation in vulnerability among FSWs with regard to police arrest. FSWs who reported being dependent solely on sex work-derived income, having higher average per-week client volume and practising sex work in public places or brothels were found to be most vulnerable to police arrest. Street-based FSWs, and particularly those with high client volumes, are generally more visible to police, which can lead to more frequent interactions with officers on the beat, leading to increased violence and harassment [54,55]. This visibility can also add to FSWs’ vulnerability to violence perpetrated by clients [56]. Similarly, given that brothels are criminalized under Section 3 of the ITPA [36], brothel-based FSWs are also particularly vulnerable to arrest and harassment by law enforcers during routine raids [57]. Previous research from Karnataka suggests that FSWs’ vulnerability to HIV varies according to typology, mainly due to disparities in programme accessibility for different types of FSWs [58]. Our findings also suggest that FSWs working away from their home district might be more isolated from social support networks and/or have limited access to services provided by FSW peer groups. As such, this group should be prioritized by interventions aiming to reduce arrest and violence.

Collectivization and empowerment of FSWs are key elements in the violence reduction strategies of *Avahan*'s HIV prevention programmes. Although sex workers collectives did previously exist in the study districts, the intervention was able to gradually create spaces where FSWs could regularly meet and share their experiences, which helped create a sense of solidarity and foster collective action [44,48]. Notably, membership in local FSW collectives increased from 11,000 in 2007 to 36,000 in 2009 [59]. In this study, we found that FSWs were more likely to report peer support following arrest in the follow-up round of IBBA surveys, suggesting that interventions with FSWs were successful in bringing them together and promoting solidarity. This finding is supported by previous work showing that FSWs who are part of peer groups have associated increases in three domains of empowerment, as conceptualized by Blanchard *et al*. [42], and experience less violence overall [51]. In particular, these peer groups provide opportunities for FSWs to meet and build a collective identity, which has been associated with reduced vulnerability to HIV [42,60].

Finally, recent modelling studies provide convincing data that support the decriminalization of sex work in order to protect the rights and well-being of FSWs and to stem HIV epidemics across diverse global contexts [2]. While advocacy and activism for decriminalization continues, creating sustainable partnerships between law enforcement bodies and FSW communities is a pragmatic and effective approach for HIV prevention programmes to ensure that FSWs have a safe working environment and adequate access to health and social services. These structural interventions need to be multilayered and multifaceted, should involve collaboration between police and FSWs and should be implemented and scaled up in parallel with effective behavioural and biomedical HIV preventive interventions [61,62].

This study has a few limitations. First, the presented data were collected and analyzed between 2010 and 2012, and the *Avahan* project was transitioned to the government of India in 2013. Despite the time that has passed since data collection, we strongly believe that our in-depth description of the processes involved in developing and implementing structural interventions with police and FSWs remains beneficial to researchers and programme implementers who are interested in rolling out HIV prevention programmes that incorporate violence reduction strategies in different contexts. Second, because the data from R2 and R3 could not be linked, our methodology is limited to comparing data over time between two cross-sectional, randomly selected samples of FSWs, rather than performing truly longitudinal analyses. However, IBBA participants were asked whether they had taken part in a previous iteration of the survey and only 5% of FSWs responding in R3 had also participated in R2 – an unsurprising finding, given the extensive mobility and migration observed among FSWs in Karnataka [63,64]. As such, the assumption of independence between R2 and R3 samples required for the multivariate logistic model used is likely appropriate. Third, because R1 (which was conducted at the same time as the structural interventions were being implemented) did not include questions about police arrest, we lack a true baseline rate of arrest with which to compare our findings in R2 and R3. Rather than assessing the interventions’ impacts on police arrest over a seven-year period, we are only able to use a 24-month study horizon. This shorter period of time may not be adequate to observe effects of slow-moving processes, such as police reform or policy change, which might further influence rates of arrest among FSWs. Finally, as noted in Table 1, the predominant method of solicitation changed significantly between R2 and R3. While similar proportions of FSWs solicited from their homes between rounds, in R3, a significantly greater proportion reported soliciting clients using mobile phones and fewer met clients in the street or other public places. The proportion of FSWs reporting ≥10 clients per week also decreased between R2 and R3. The adjusted odds ratios obtained from our multivariate analyses, presented in Table 4, have been controlled for these profile changes, thus minimizing the likelihood that they are contributing to the observed reductions in police arrest over time. As such, our findings clearly emphasize the need for structural interventions focused on reducing violence to prioritize services that reach FSWs who are disproportionately vulnerable to police arrest.

## Conclusions

The findings from our study provide additional evidence that structural interventions are useful components of broader strategies to reduce the rates of police arrest among FSWs. Specifically, targeting structural interventions to police is a novel approach that should be considered in contexts outside of South India, in which state actors are common perpetrators of violence against FSWs. Importantly, programmes aimed at reducing violence, including arrest, among communities of FSWs have been shown to reduce the incidence of HIV among those disproportionately vulnerable to infection, including FSWs [1,3,6,17]. In addition to effective behavioural and biomedical interventions, structural interventions that incorporate police training and sensitization, as well as capacity building and collective empowerment strategies for FSWs, must be included in comprehensive HIV prevention programming for FSWs.
